# Rare earth nanoparticles for sprayed and intravenous NIR II imaging and photodynamic therapy of tongue cancer†

**DOI:** 10.1039/d2na00197g

**Published:** 2022-04-11

**Authors:** Lingling Cai, Zhan Wang, Bi Lin, Kaikai Liu, Yanxing Wang, Ying Yuan, Xiaofeng Tao, Ruichan Lv

**Affiliations:** Department of Radiology, School of Medicine, Shanghai Ninth People's Hospital, Shanghai Jiao Tong University Shanghai 200011 China yuany83@163.com cjr.taoxiaofeng@vip.163.com; Engineering Research Center of Molecular and Neuro Imaging, Ministry of Education, School of Life Science and Technology, Xidian University Xi'an Shaanxi 710071 China rclv@xidian.edu.cn

## Abstract

In this research, rare earth nanoparticles coupled with dihydroartemisinin (DHA) and a targeted antibody (RENP-DHA-Cap) for sprayed NIR II imaging and photodynamic therapy (PDT) of tongue cancer were designed. Genetic algorithms combined with combinatorial chemistry were proposed and successfully achieved in a single optimized luminescent phosphor with enhanced NIR II and high upconversion luminescence (UCL) under a NIR laser of wavelength 980 nm or/and 808 nm. In particular, T1 magnetic resonance imaging (MRI) signals can be adjusted with the Gd ion concentration. In combination with the targeted antibody of capmatinib (Cap), precise NIR II imaging for *in situ* tongue cancer by a simple spray method can be achieved. Most importantly, NIR II imaging and PDT treatment can be realized with RENP-DHA-capmatinib injected intravenously. This orthogonal theranostic mode with precise diagnosis under 808 nm and targeted effective treatment under 980 nm may promote tongue cancer theranostics.

## Abstract 2

In this research, rare earth nanoparticles coupled with dihydroartemisinin (DHA) and a targeted antibody (RENP-DHA-Cap) for sprayed NIR II imaging and photodynamic therapy (PDT) of tongue cancer were designed.

## Graphical abstract

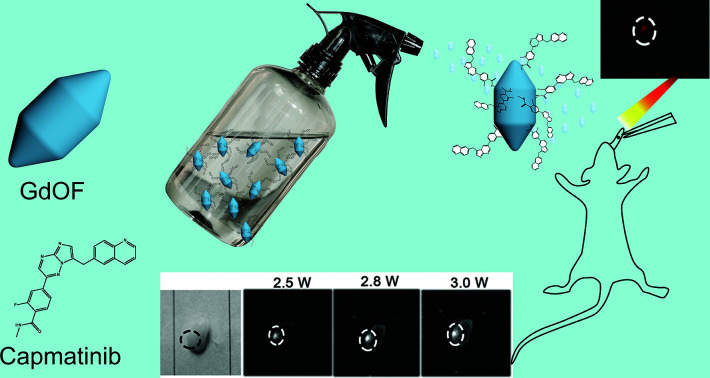

## Introduction

Molecular imaging has been widely used in disease diagnosis and treatment, and plays an important role in the field of clinical medicine. Traditional imaging methods generally use visible light to excite dye probes. Due to the absorption of visible light by tissues, the imaging effect of the visible light excitation probe was often not satisfactory.^1–3^ NIR II imaging (1000–1700 nm) has been proposed and characterized by low tissue absorption, low autofluorescence background, excellent optical stability, high resolution, high sensitivity, high penetration depth, and weak tissue light damage, which is very suitable for disease imaging and diagnosis.^4–11^ Rare earth-doped nanoparticles (RENPs) are generally composed of a host matrix and an activator, which are inorganic photoluminescent materials with various emission bands.^12^ The sensitizer is doped together with the host matrix and the activator, which can enhance and transfer the absorbed energy to the activator to improve the energy transfer efficiency. In past research, metal modulation,^13–17^ codopant adjustment,^18–21^ dye sensitization,^22–26^ multi-level structure^27–30^ and other methods have been used to improve the luminous intensity. For multi-element-doped luminescent powders, we have used a heuristic algorithm to effectively search for brighter luminescent powders based on the brightest luminescent powders available.

Photodynamic therapy (PDT) has been widely studied and clinically used for cancer therapy especially in the assisted therapy field due to its advantages of non-invasiveness, less side effects, and strong tumor targeting.^31–34^ The PDT process involves excitation of a photosensitizer by light, which then reacts with oxygen to produce reactive oxygen species (ROS).^35,36^ Artemisinin contains peroxygen bonds widely used for malaria treatment. During the anti-malarial process, free radical groups will be generated in the body to combine with the plasmodium protein and change the cell membrane structure of the plasmodium. Artemisinin produces free radicals and affects the surrounding cells in a similar way to PDT photosensitizers. Other experimental studies have shown that dihydroartemisinin (DHA) also has PDT effects.^37,38^ However, the absorption wavelength of artemisinin belongs to visible light, and it does not penetrate the tissues sufficiently.^39^ Therefore, up-conversion nanoparticles with anti-Stokes energy transfer (long-wavelength excitation and short-wavelength emission) can be proposed to improve their penetration.^40,41^ The RENP has both up-conversion and down-shifting NIR II imaging properties under the irradiation of near-infrared light, with deep penetration, low autofluorescence, and high imaging signal-to-background ratios.^42–47^ Therefore, RENPs are very suitable as energy donors in combination with DHA as diagnostic and therapeutic reagents.

In this study, a co-precipitation method was used to synthesize RENPs. In order to optimize the NIR II luminescence, genetic algorithms were used to guide the optimization of the doping composition (Yb, Er, Ce, and Eu) of RENPs. The NIR II imaging and the UCL of different generations of RENPs were compared. The T1 magnetic resonance imaging (MRI) signal can be adjusted with the Gd ion concentration. After optimization, the luminescent powder with both strong UCL visible light emission under 980 nm and NIR II imaging under 808 nm laser can be selected for diagnosis and treatment separately. The RENP was coupled with DHA to form a RENP-photosensitizer compound, and the compound was tested for the PDT effect. In addition, the RENP was also applied to *in situ* NIR II imaging by spraying and to the treatment of tongue cancer *via* the tail vein.

## Experiments and methods

### Chemicals and reagents

Gd(NO_3_)_3_ (99.99%), Yb(NO_3_)_3_ (99.99%), Er(NO_3_)_3_ (99.99%), Ce(NO_3_)_3_ (99.99%), Eu(NO_3_)_3_ (99.99%), urea, dihydroartemisinin (DHA), potassium fluoride (KF), and PAA (polyacrylic acid) were obtained from Shanghai Aladdin Bio-Chem Technology Co., Ltd, Shanghai, China. All chemical reagents were used as received without further purification. Cal27 cells (human tongue squamous cell carcinoma) were purchased online (https://www.atcc.org/, USA).

### Synthesis of RENPs

The synthesis of GdOF was taken as an example. The synthesis was performed following the procedure reported in our past work, with slight modifications.^48,49^ Typically, 1 mmol of gadolinium nitrate and 1.5 g of urea were added to a beaker. Then, 0.1 g of KF was added under magnetic stirring. After stirring for 5 minutes, it was put in a water bath at 90 °C for 3 h. After that, the solution was transferred into a centrifuge tube and centrifuged at 6000 rpm for 6 min to obtain a phosphor precipitate. Finally, the precipitate was washed 3 times with ultra-clean water and placed in an oven at 60 °C for drying. Finally, the dried precipitate was put into a crucible, which was placed inside a muffle furnace and sintered at 500 °C for 3 h to obtain GdOF. The corresponding amounts of Yb^3+^, Er^3+^, Ce^3+^, and Eu^3+^ with the total amount of 1 mmol were added to obtain RENPs with different codopant concentrations.

### Genetic algorithm optimization

The genetic algorithm reported in our past work was used with slight modifications.^50,51^ In this study, we studied RENP phosphors coped with Yb, Er, Ce, and Eu for NIR II downshifting luminescence under the single 808 nm NIR excitation source and UCL under the 980 nm laser. The steps of the genetic algorithm in this article are as follows: (1) The volume of each component of 20 samples was taken as the parent data, the NIR II imaging of the RENP was measured, and the luminescence intensity was used as the fitness of the algorithm. Note that there should be no brightness overexposure in the data that need to be processed by the algorithm. Overexposure will affect the real luminescence brightness collection and increase the error. (2) The fitness of last generation was referenced; the higher the fitness, the greater the probability of being selected. In the process of elitism, the code with the highest paternal fitness was directly replaced with the code of the next generation. (3) Two parent codes were selected for single-point crossover, and then two new codes were generated. (4) The newly generated code was subjected to a single point mutation operation. (5) The generated new code was normalized to get two progeny codes. (6) The above-mentioned steps were repeated until 20 newly generated codes were generated as the next-generation synthetic data. After the composition and dosage of the new generation of luminescent powder were known, the new generation of luminescent powder was synthesized according to the above-mentioned co-precipitation method. Then, we collected data and optimized using genetic algorithms until the maximum evolutionary generation number was obtained. In this research, these processes combined with experiments and genetic algorithms include the synthesis of phosphors, brightness evaluation, and optimized calculation using brightness as the fitness reference, and the new composition calculation was carried out to find the brightest Yb, Er, Ce, and Eu-doped phosphors. By repeating these processes for four generations (with one added half-generation in the first generation to ensure the brightness accuracy), the new phosphors of the next generation are guided by the algorithm calculation results and experiments.

### Synthesis of RENP-DHA and RENP-DHA-Cap

Typically, 50 mg of RENP with strong red UCL light under 980 nm laser and 50 mg of PAA (polyacrylic acid) were placed in a small beaker. Then, 20 mL ultrapure water was added to dissolve ultrasonically and stirred for 12 hours. It was centrifuged at 7500 rpm for 7 min and dissolved in water. Then, 30 mg of *N*-(3-dimethylaminopropyl)-N′-ethylcarbodiimide hydrochloride (EDC) powder and 90 mg of *N*-hydroxysulfosuccinimide (NHS) powder were added and stirred for half an hour. Second, 50 mg of DHA and 500 μL chloroform were added to obtain a DHA solution under dark conditions. It was stirred for 12 h in a small beaker, and the mixture was centrifuged at 6500 rpm for 10 min. Finally, the RENP-DHA sample was obtained and stored away from light. For RENP-anthocyanin as the control PDT sensitizer, DHA was replaced with anthocyanin, which was obtained from the flowers by simple concentration through centrifugation after being broken by grinding according to our previous research.^52^

A small beaker containing a RENP-DHA solution was placed on a stirrer, and 30 mg of EDC powder and 90 mg of NHS powder were added and stirred for half an hour. Then, 50 μL of capmatinib (1 mg mL^−1^) was added in the darkness and stirred on ice overnight. Then, the mixture was centrifuged at 6500 rpm for 10 min, and then the RENP-DHA-Cap sample was obtained. The obtained RENP-DHA-Cap was stored in a fridge at 20 °C for a short period (less than one week) or at −80 °C for a long period (less than one year) in order to keep the active drug efficiency.

### DPBF test for the PDT effect in tubes

Diphenylisobenzofuran (DPBF) is an organic fluorescent dye, and DPBF can react with reactive oxygen species (ROS). Here, we use DPBF to detect the PDT effect in the tubes with different irradiation time points. During the reaction, the color of DPBF will gradually become lighter with the extension of the reaction time, and the absorption at 400 nm will gradually decrease. Therefore, the absorption change of DPBF at 400 nm can be detected under different light time points to reflect the amount of produced ROS, thereby indicating the PDT effect.

### Targeting effect of RENP-DHA-Cap

Cal27 cells were co-incubated with RENP-DHA and RENP-DHA-Cap for different time periods (1 h, 3 h, and 5 h, respectively). In another phagocytic blocking experiment, Cap was added prior to co-incubation. Typically, Cal27 cells were also co-incubated with RENP-DHA-Cap for different time periods (1 h, 3 h, and 5 h, respectively). In addition, nuclei were labeled with DAPI before imaging using a fluorescence microscope.

### Calcein-AM/PI staining

RENP-DHA and RENP-DHA-Cap were co-incubated with Cal27 cells for a period of 3 h, followed by 980 nm laser irradiation (0, 1, 3, 5 min) and no irradiation. Calcein-AM (1 mL, 5 μM) and PI (1 mL, 5 μM) were used for staining, and then, the cells were observed using a fluorescence microscope. Calcein-AM stained only live cells and PI dye could not cross the membrane of live cells, and therefore, under the microscope, green fluorescence represents living cells and red fluorescence represents dead cells.

### Biocompatibility of RENP-DHA-Cap by CCK-8 assay

The toxicity of RENP-DHA-Cap was evaluated by a CCK-8 assay on tumor cells and normal cells. Tumor and normal cells were incubated in a 96-well plate in an atmosphere containing 5% CO_2_ at 37 °C for 12 h. Different concentrations of 10 μL RENP-DHA-Cap (250, 125, 62.5, 31.3, and 15.6 μg mL^−1^) were then added to the 96-well plate. After incubation in an atmosphere containing 5% CO_2_ at 37 °C for 12 h, the separate Cal 27 tumor cells or normal cells were washed several times with 100 μL PBS and then 200 μL medium and 10 μL of CCK-8 solution (5 mg mL^−1^) were added to each well. Note that here the experiments should be carefully carried out in order to avoid bubbles in the wells, which may affect the absorbance value. Then, the 96-well plate was incubated for further 4 h. Finally, the absorbance at 450 nm was measured using a microplate reader.

### *In vivo* NIR imaging

All animal procedures were performed in accordance with the Guidelines for Care and Use of Laboratory Animals of Shanghai JiaoTong university and approved by the School of Pharmacy’ s Ethics Committee of Shanghai Ninth People's Hospital, Shanghai JiaoTong university (SH9H-2020-A620-1). Cal27-Luc cells were growing for subcutaneous tumors and *in situ* tumors in the tongue. The NIR II imaging of a mouse with *in situ* tumors was performed after treatment with the spray of RENP-DHA-Cap. The NIR II imaging of a mouse with subcutaneous tumors was performed after tail vein injection of RENP-DHA-Cap.

### *In vivo* tumor treatment

Cal27-Luc cells were growing for subcutaneous tumors. When the tumor grew to a size of 0.1–0.15 cm^3^, a tumor-bearing mouse model was successfully established. Nine mice were divided into 3 groups: the “RENP + Laser” group was injected with RENPs (100 μL, 1 mg mL^−1^) through the tail vein, the “RENP-DHA-Cap + Laser” group was injected with RENP-DHA-Cap (100 μL, 1 mg mL^−1^) through the tail vein, and the control group was without treatment. The tumor was irradiated with a NIR laser at 1.0 W cm^−2^ for 10 min. Treatment took place every two days, and seven treatments were processed (14 days). The body weight and tumor size of the mice were also recorded during treatment.

## Results and discussion

### Genetic algorithm optimization of RENPs and related characterization

**Scheme 1:**
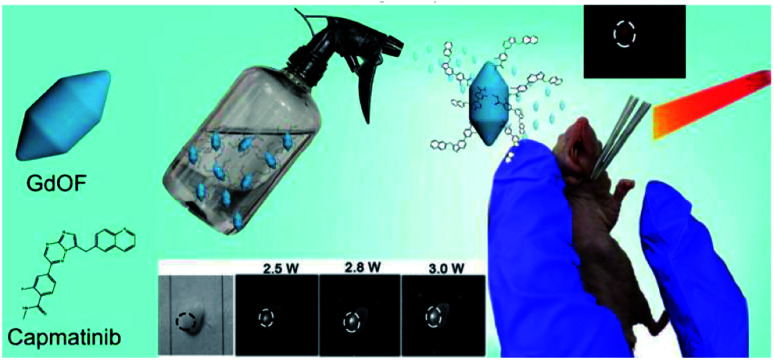
Schematic diagram of the designed nanoparticles and the biological use for tongue cancer luminescence imaging (by spray).

The schematic diagram shows the designed nanoparticles for cancer imaging (Scheme 1). Typically, RENPs coupled with dihydroartemisinin (DHA) and targeted antibody (RENP-DHA-Cap) for sprayed NIR II imaging and photodynamic therapy (PDT) of tongue cancer was designed. The genetic algorithm is used to guide the concentration of the sensitizer and the activator. The optimized RENP is coupled with DHA to obtain a compound with excellent *in vivo* NIR II luminescence intensity under 808 nm irradiation and photodynamic therapy (PDT) under 980 nm irradiation. Moreover, the targeted property was obviously enhanced due to the recognition of capmatinib to the cMET protein in the tumor cells. Besides *in vitro* experiments, *in vivo* theranostic experiments including spray imaging and intravenous imaging/therapy were carried out.

**Fig. 1:**
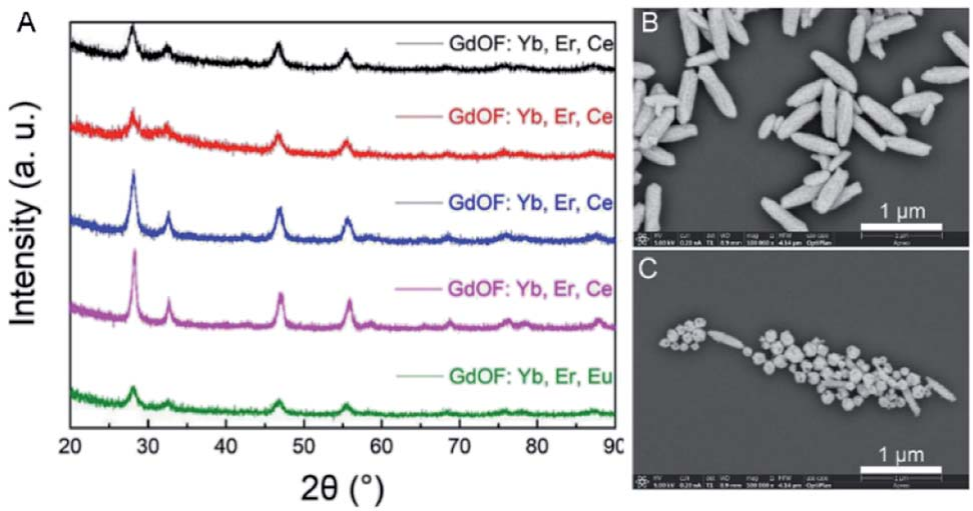
(A) XRD pattern of five rare earth nanoparticles. The concentration lists in (A) shown from top to bottom are: 0.1Yb,0.01 Er,0.02Ce; 0.1Yb,0.01 Er,0.05Ce; 0.2Yb,0.01 Er,0.02Ce; 0.2Yb,0.02 Er,0.02Ce; 0.1Yb,0.01 Er,0.02Eu. SEM images of (B) no. 4 RENP and (C) no. 14 RENP in the fourth generation.

In this study, the RENP was synthesized by a co-precipitation method. First, the influence of doping of different elements on the RENP lattice was studied according to the controlled variable method. After a batch of samples were synthesized, the X-ray diffraction (XRD) measurement was carried out. Fig. 1A shows the XRD pattern of the RENP with five different element ratios, indicating that the doping changes of Yb, Er, Ce, and Eu did not have a significant impact on the crystal lattice of the nanoparticles. It ensured that the genetic algorithms proceed without considering the effect of the lattice on the optimization effect. Moreover, the morphology can be kept uniform with different codopants (Fig. 1B and C). Here, we chose the samples of no. 4 and no. 14 in the fourth generation because these two samples have obvious codopant differences. For no. 4, the Gd concentration is 0.61; for no. 14, the Gd concentration is 0.36. When the Gd concentration is higher, the spindle-like nanoparticles are more.^48,49^ Yet, the nanoparticles remain monodisperse no matter what the codopant is.

**Fig. 2:**
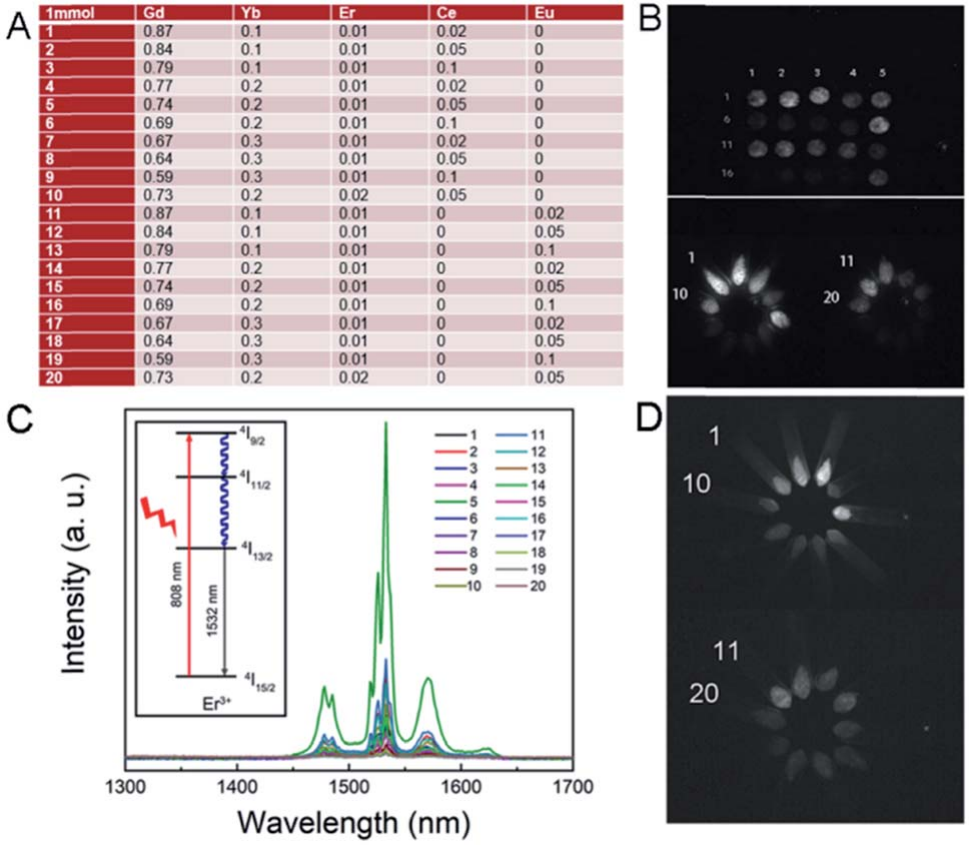
(A) Proportion of the RENP sample in the first generation. (B) NIR II imaging of the RENP (808 nm, 0.4 W, LP 1300). (C) Corresponding NIR II luminescence spectra. The inset is the energy transfer process of NIR II emission under a 808 nm laser using the energy levels. (D) NIR II imaging of the RENP (980 nm, 0.5 W, LP 1300).

After determining the proportion of each component of the first-generation RENP (Fig. 2A) and the amount, the first-generation RENP was synthesized by a co-precipitation method, and we performed NIR II imaging on it. As shown in Fig. 2B and C, according to the analysis of luminescence intensity and composition ratio, the following conclusions can be drawn preliminarily: (1) under 808 nm excitation, the luminescence intensity of Ce doping was mostly stronger than that of Eu doping. (2) The luminescence intensity with different Yb concentrations was compared: 10% > 20% ≈ 30%. Thus, when Yb accounted for 10%, the brightness was the highest. (3) Luminescence intensities were compared at different Er concentrations: 2% > 1%, hence the brightness was highest when Er took 2%. (4) When the Yb concentration was designed at 10% and the Er concentration was at 1%, the relationship between the proportions of Ce and the luminous intensity was 2% < 5% < 10%, while the proportions of Yb are 20% and 30%, and when Er is at 1%. The relationship of different proportions of luminous intensity was 2% > 5% > 10%. The reason for these results is that the Yb sensitizer and Ce sensitizer were utilized to adjust the NIR II imaging and the total amount of Yb/Ce should be stable. (5) When the proportion of Eu was different and that of the other elements were kept the same, the relationship of luminous intensity was 2% > 5% > 10%, as shown in Fig. S1.† Compared with the proportions of the components of the supplementary experiment and the luminous intensity of the NIR II, the following conclusions can be drawn through analysis: the relationship between the concentration of Yb and the luminous intensity is 10% = 5% < 8%. The extreme value was 8% for the Yb dopant; the luminescence intensity of the Yb group was lower than that of the Gd group. Other laws were consistent with the first generation.

Note that here we used 808 nm as the excitation laser for NIR II imaging. In most of the past research on the RENP, researchers found that 808 nm has less photothermal toxicity to the normal cell than that of the 980 nm laser.^53,54^ Then, we designed the samples with 808 nm laser for imaging and separate 980 nm for up-conversion guided PDT. Indeed, in this research, the RENP doped with Yb, Er, Ce, and Eu shows NIR II imaging under 980 nm. The NIR II imaging photographs of RENP samples under a 980 nm laser are shown in Fig. 2D to study the multi-spectra properties. As shown, the chosen samples can have good NIR II imaging under 980 nm laser, although the brightest value occurs in different samples because of different energy transfer processes from 808 nm or 980 nm. As shown, the main NIR II imaging property (LP1300) was generated from the 1532 nm peak of Er^3+^ ions.

**Fig. 3:**
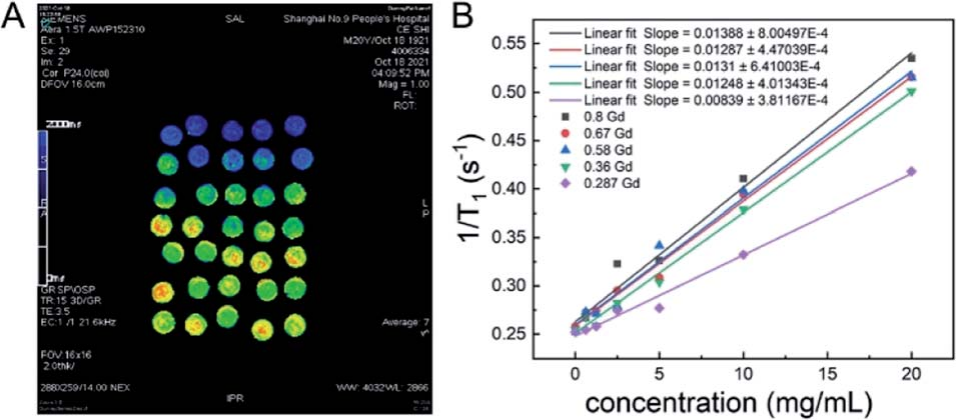
(A) *T*_1_ mapping images of MRI, and (B) the relationship between *T*_1_ relaxation rates and the sample concentration at different Gd concentrations.

Then, under the guidance of genetic algorithms, the optimization was performed on the basis of the first-generation RENP, and the percentage data of each element of the second-generation to the fourth-generation RENP were obtained. The RENP was also synthesized by the co-precipitation method, and the NIR II imaging of the samples from second-generation to the fourth-generation RENP was performed (Fig. S2–S4†). At the same time, we acquired the MRI mapping images of different Gd concentrations (Fig. 3). It showed that the RENP had a shorter T1 relaxation time and a higher relaxation rate (slopes increased from 0.0084 to 0.0139) with a higher Gd concentration (Gd concentration from 0.287 to 0.8). Note that some of the data are not totally linear, and it may be attributed to the high concentration-introduced aggregation (highest concentration: 20 mg mL^−1^), which reduced in a partially heterogeneous magnetic field.

### Photoactive effect of RENP-DHA

**Fig. 4:**
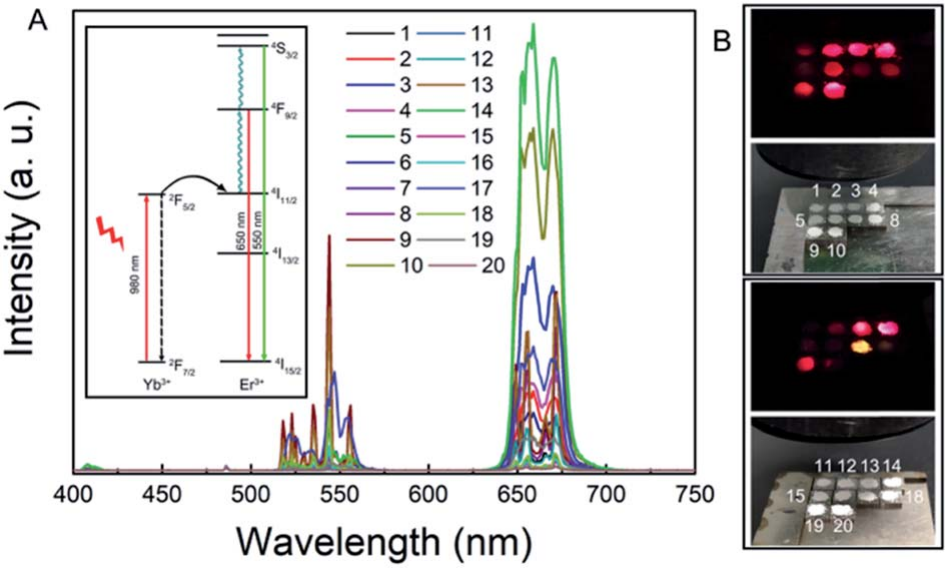
(A) UCL spectra and (B) UCL light of the RENP samples in the fourth generation under 980 nm excitation. Inset is the energy transfer process of UCL under 980 nm laser using the energy levels.

**Fig. 5:**
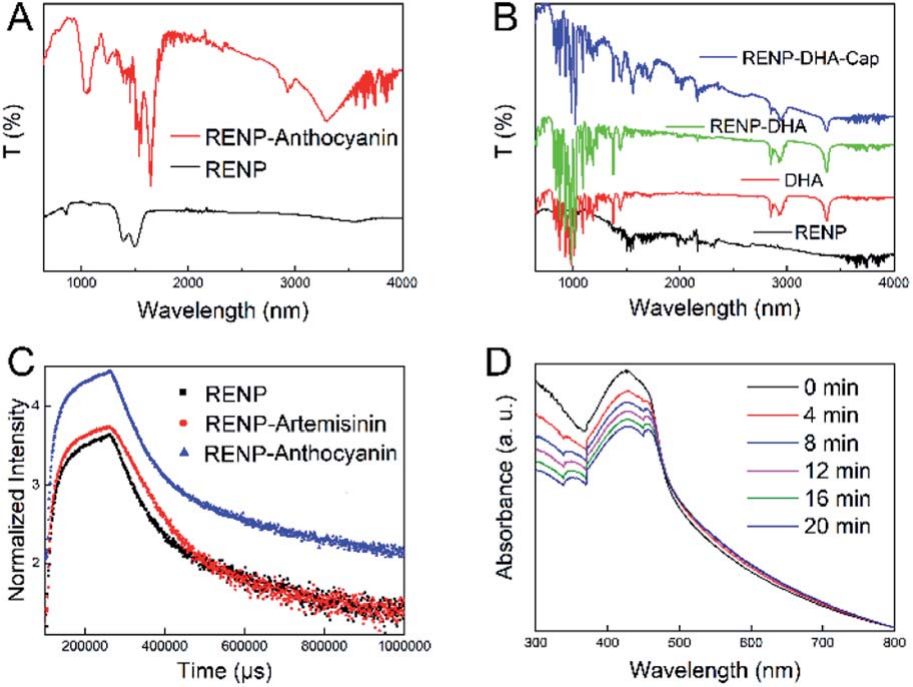
(A) FTIR patterns of RENP and RENP-Anthocyanin. (B) FTIR patterns of RENP, DHA, RENP-DHA, and RENP-DHA-Cap. (C) Lifetime curves of RENP, RENP-DHA, and RENP-Anthocyanin (emission: 654 nm; excitation: 980 nm). (D) UV-vis absorbance of DPBF solution together with RENP-DHA under 980 nm laser with different irradiation time periods (0–20 min).

In the above-mentioned experiment, we had obtained the RENP with the highest NIR II luminescence intensity. The RENP was irradiated with a 980 nm laser and it was found that most of the RENP emit strong red UCL light (Fig. 4). Then, the RENP which had both good down-shifting NIR II luminescence intensity under 808 nm excitation and bright red UCL emission under 980 nm laser irradiation was selected. The optimized sample was coupled with two photosensitizers: anthocyanin (as a control) and DHA. The FTIR spectra (Fig. 5A and B) showed that anthocyanin and DHA can be successfully linked on the surface of the RENP. Moreover, the TEM image of RENP-DHA-Cap shows that there is a hydrous shell coated on the surface of the RENP (Fig. S5†). In addition, the particle size and zeta potential of the RENP, RENP-DHA and RENP-DHA-Cap were measured. As shown in Fig. S6,† the particle sizes of the RENP, RENP-DHA and RENP-DHA-Cap were 314.8 nm, 308.0 nm and 313.2 nm, respectively. These results indicate that the RENP was uniform and stable. The zeta potential values (Fig. S7†) of the RENP, RENP-DHA and RENP-DHA-Cap were 40.3 mV, 37.0 mV and 35.1 mV, respectively. In addition, the zeta potential of RENP-PAA is −24.4 mV. These results indicate that DHA and capmatinib were well conjugated with the RENP. The lifetimes of the RENP, RENP-DHA, and RENP-anthocyanin were 0.076 ms, 0.061 ms, and 0.073 ms (Fig. 5C), respectively. According to the light conversion efficiency formula: *E* = 1 − *τ*_DA_/*τ*_D_, the conversion efficiency of the RENP for artemisinin and anthocyanins was 19.3% and 3.4%, respectively. Therefore, RENP-DHA can be used as a photosensitizer under near-infrared light irradiation for PDT cancer therapy.

DPBF was further used to indicate the ROS generation under 980 nm laser. After adding the DPBF solution, the solution was exposed to a 980 nm laser for 2 min each time, and the solution was measured using an ultraviolet spectrophotometer to analyze the PDT effect. Fig. 5D shows that the absorption of the solution at 400 nm gradually decreased with the prolonged illumination time, indicating the existence of ROS when the RENP-DHA complex was irradiated under 980 nm excitation. It further proved that the complex had a certain PDT effect. When compared with the ROS detection results of anthocyanin (Fig. S8†), it was found that the RENP-DHA complex made the absorption of DPBF at 400 nm drop faster, indicating that it can produce more ROS and have a better PDT effect. At the same time, we verified the photothermal effect of RENP-DHA. As shown in Fig. S9,† the synthesized RENP-DHA has a slight photothermal effect. The temperature difference in the tubes (solution volume: 2.5 mL) between the PBS and RENP-DHA is 5.5 °C, and the temperature difference in the tumor focus of the mice is 9.8 °C. The reason for this slight photothermal effect is that the absorbed upconversion luminescence from the RENP to DHA was mainly for PDT effects instead of photothermal therapy (PTT) effects.

### *In vitro* targeting and PDT effect

**Fig. 6:**
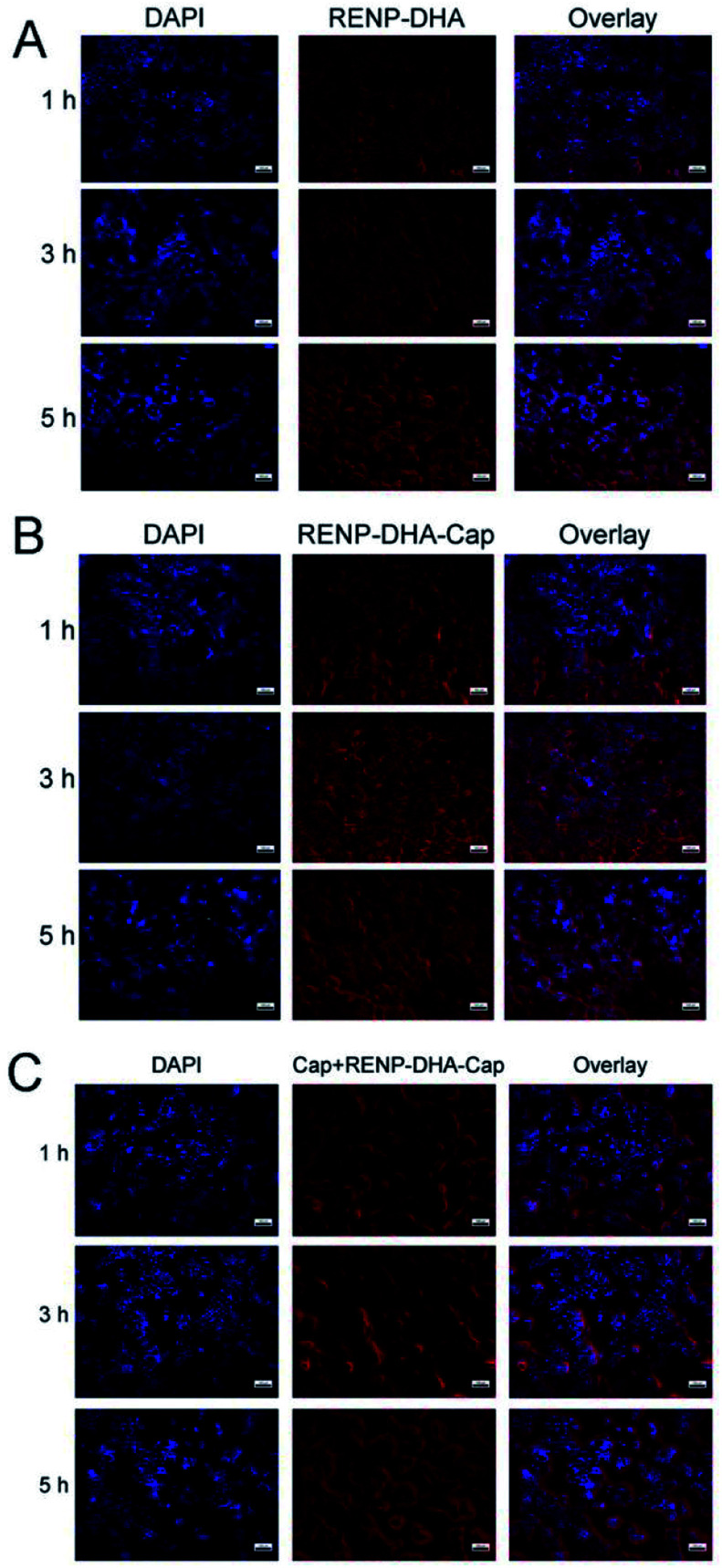
Microscopic images of Cal27 cells co-incubated for 1 h, 3 h, and 5 h with (A) RENP-DHA group, (B) RENP-DHA-Cap group, and (C) blocked Cap + RENP-DHA-Cap group. The scale bar is 100 μm in all the figures. Here, the blue channel means the DAPI as the control (nuclei marked); the red channel means the fluorescence of the RENP (cytoplasm marked).

Since human-derived head and neck squamous cell carcinoma (Cal27 cell line) expresses c-Met, capmatinib in RENP-DHA-Cap can target c-Met. First, the experiment proved the biocompatibility of RENP-DHA and RENP-DHA-Cap *in vitro*. Cal27 cells were co-incubated with RENP-DHA and RENP-DHA-Cap for 1 h, 3 h, and 5 h respectively and stained with DAPI, and the phagocytic effect of cells can be observed under a fluorescence microscope (Fig. 6A and B). At the same time, we performed a block phagocytosis experiment. After adding Cap first to the Cal27 cells, the cells were co-incubated with RENP-DHA-Cap for different time periods (Fig. 6C). As shown in Fig. 6A and B, cells can phagocytose RENP-DHA and RENP-DHA-Cap well, and RENP-DHA-Cap shows better targeting effects on cells. In the block phagocytosis experiment, the intensity of red fluorescence was weakened, which proved that the added Cap would have a certain blocking effect on the phagocytic material (RENP-DHA-Cap) in the cell. In Fig. 6, the red/blue ratio of the three materials at 3 h was calculated using ImageJ, which was used to indicate the intracellular effect. In addition, the red/blue ratio values of RENP-DHA, RENP-DHA-Cap, and the blocked Cap + RENP-DHA-Cap are 0.82, 1.44, and 0.93, respectively. These results proved the good targeting effect of capmatinib to Cal 27 tumor cells.

Furthermore, the ROS amount *in vitro* was detected. We used the DCFH-DA to marked the ROS amount after the RENP-DHA-Cap intracellular by the Cal 27 cells. The microscopic images of Cal 27 cells incubated with RENP-DHA-Cap under a 980 nm laser with different irradiation time periods (0, 3, 5, and 7 min) are shown in Fig. S10.† All the cells were finally marked with DCFH-DA. As shown, the ROS amount increased with the prolonged irradiation time.

In the Calcein-AM/PI staining experiment, live cells showed green color and dead cells showed red color. Fluorescence microscopic images of Cal27 cells that were incubated with the materials were recorded. Fig. S11† and 7 show the fluorescence microscopic images of cells co-incubated with RENP-DHA and RENP-DHA-Cap when a 980 nm laser was irradiated for 0, 1, 3, and 5 min, respectively. Moreover, the green/red ratio was calculated using ImageJ in order to indicate the live/dead state. As shown, the RENP-DHA-Cap has higher and faster PDT effects to kill the tumor cells *in vitro*. These results indicate that the material has good biocompatibility. After irradiated with a 980 nm laser, the red fluorescence increased and the green fluorescence decreased. These results indicated that the material had a significant PDT effect.

**Fig. 7:**
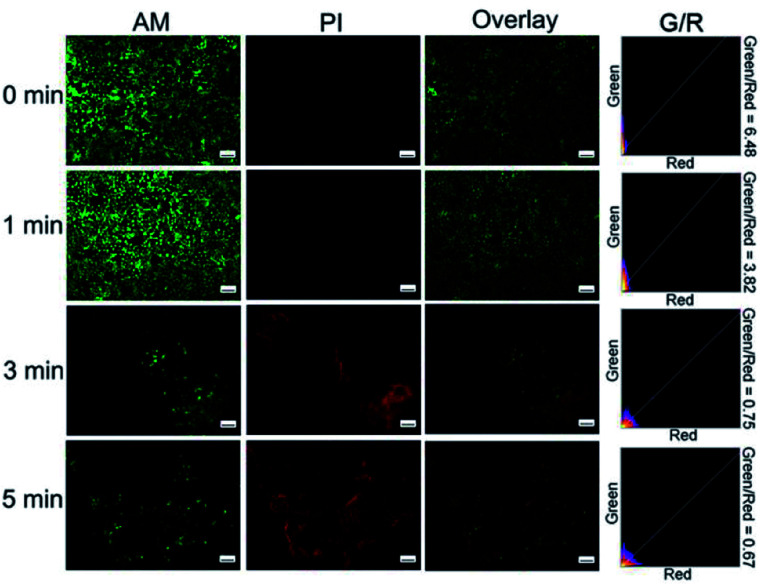
Microscopic images of Cal27 cells co-incubated with RENP-DHA-Cap for 0, 1, 3, 5 min under 980 nm irradiation (stained with Calcein-AM/PI). The scale bar is 100 μm in all the figures.

### *In vivo* imaging and treatment effect

The RENP synthesized in this experiment had a superior NIR II and UCL imaging effect. We coupled it with DHA for PDT, and loaded the Cap to enhance the targeting effect. As mentioned above, the RENP with different Gd concentrations (0.287–0.8) has the effect of MRI, and then we simply performed *in vivo* MRI using three samples injected subcutaneously (Gd concentrations in the RENP: 0.36–0.67; RENP concentration in PBS: 5 mg mL^−1^). The overall MRI signal after injection of RENP samples can be observed in Fig. S12.† Then, the *in vivo* imaging and therapeutic experiments were carried out. First, we studied the accumulation of particles *in vivo* in order to see the long-term toxicity. As shown in Fig. S13,† the nanoparticles are mainly located in the liver, spleen, and kidney at 6 h and the accumulation amount decreased within 7 days. Due to the experiment limitation, other long-time toxicity experiments were not carried out. Yet, in the past research, we have conducted the experiments of the *in vivo* distribution and the excretion methods of lanthanide nanoparticles in detail together with the blood routine.

**Fig. 8:**
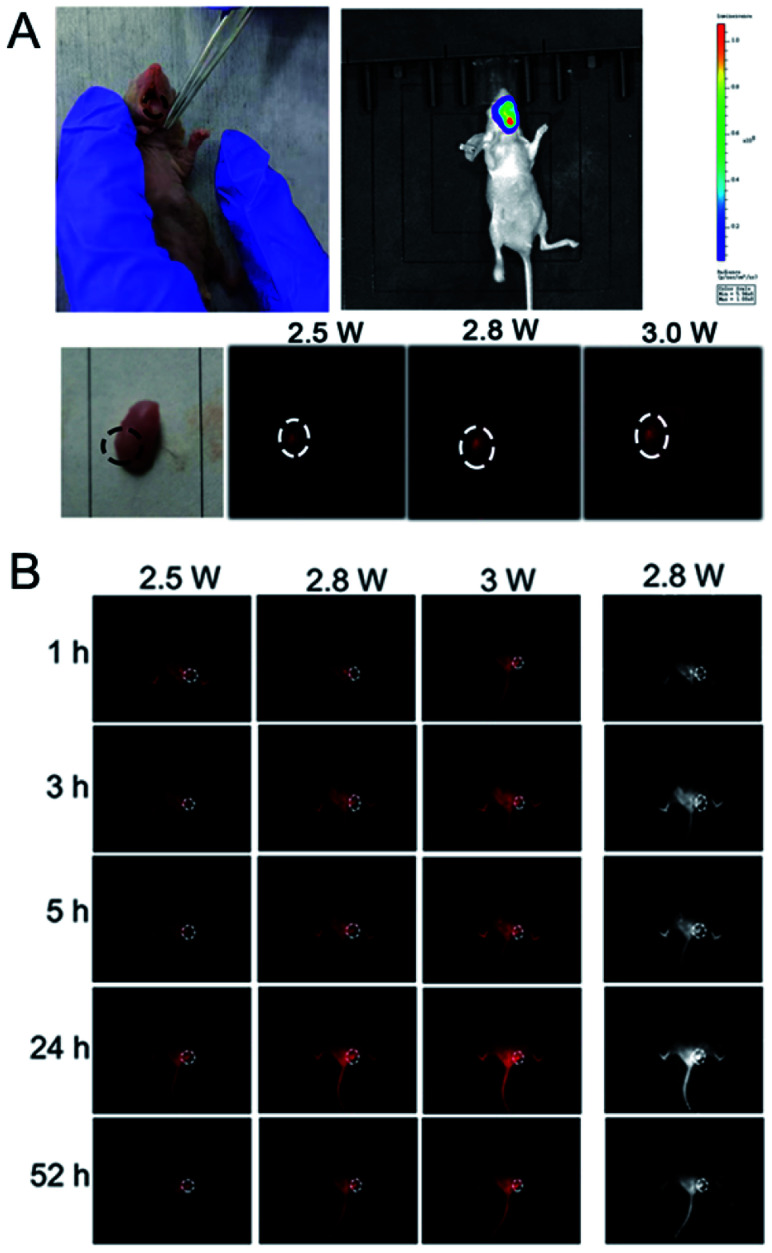
(A) NIR II imaging of a mouse with *in situ* tumors after treatment with the spray of RENP-DHA-Cap. (B) NIR II imaging of a mouse with subcutaneous tumors in the tongue after tail vein injection of RENP-DHA-Cap. Here, when the pump power was 2.8 W, the corresponding power density was about 0.3 W cm^−2^.

RENP-DHA-Cap was injected into mice by tail vein injection. The NIR II imaging of the mouse with *in situ* tumors in the tongue is shown in Fig. S14.† As known, the tongue tissue is loose and has a rich blood circulation and lymphatic drainage system, so the sprayed materials were easy to gather on the tumor site. Therefore, after a mouse with *in situ* tumors was sacrificed in the experiment, the tongue was removed and the RENP-DHA-Cap was used for spraying. After washing twice, NIR II imaging was performed and presented strong NIR II imaging at the tumor focus (Fig. 8A). In addition, the NIR II imaging of the mouse with subcutaneous tumors is shown in Fig. 8B. At 24 h, the NIR II imaging of subcutaneous tumor sites was significantly concentrated. The signal value at the tumor in the figure was significantly higher than that of the normal tissue, indicating that the material was targeted at the tumor site.

**Fig. 9:**
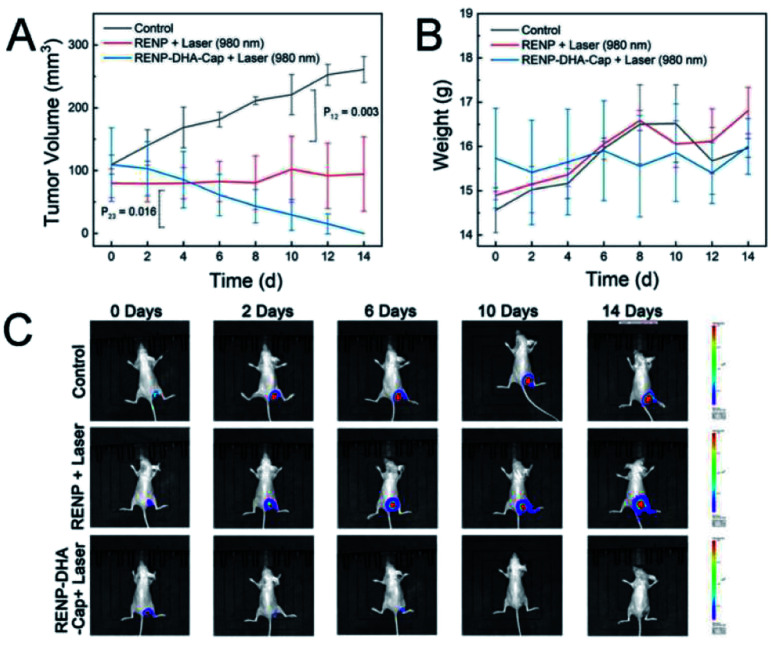
(A) Change curves of the tumor volume and (B) body weight during the 14 day treatment. (C) Bioimaging pictures of Luc-labeled Cal27 tumor cells in mice. The “RENP + Laser” group was injected with RENPs through the tail vein, the “RENP-DHA-Cap + Laser” group was injected with RENP-DHA-Cap through the tail vein, and the control group was without treatment.

Finally, nine mice were randomly divided into three groups: the control group without any treatment (the first group), the 980 nm laser irradiation treatment group after the injection of RENPs (the second treatment group), and the 980 nm laser irradiation treatment group after the injection of RENP-DHA-Cap (the third treatment group). Fig. 9A and B show the tumor volume and mice weight, respectively. In Fig. 9A, the *P* value between the control group and the second treatment group is 003 (significantly different), and the *P* value between the second and third treatment groups is 0.016 (different). The best treatment group is the RENP-DHA-Cap with NIR laser, and all the weight values kept increased. The bioimaging of the Luc-labeled tumor cells is shown in Fig. 9C, which indicates that RENP-DHA-Cap under 980 nm laser irradiation has a very good therapeutic effect, and the tumor was almost completely ablated after 14 days of treatment.

## Conclusions

All in all, this study used genetic algorithms to optimize the luminescence of RENPs in NIR II imaging. The optimized RENP was developed with both strong NIR II luminescence under 808 nm laser and red UCL light under 980 nm laser. In particular, the T1 MRI signal can be adjusted by the Gd ion concentration. When the RENP is coupled with DHA, it can have a good PDT effect. In addition, by synthesizing RENP-DHA-Cap, the probe has certain targeting ability. Through *in vivo* and *in vitro* experiments, this RENP-DHA-Cap probe can be used for *in situ* imaging and treatment of tongue cancer, providing new ideas and prospects for the diagnosis and treatment of human tongue squamous cell cancer.

## Consent for publication

All authors agreed to publish this manuscript.

## Data availability

All data generated or analyzed during this research are included in this published article and its additional information files

## Author contributions

L. C., Z. W., and B. L. performed the synthesis, MRI imaging, and *in vivo* experiments; Z. W., B. L., K. L. and Y. W. performed the synthesis, cell experiments, and NIR II imaging experiments; L. C. and Z. W. wrote the manuscript; Y. Y., X. T., and R. L. revised the manuscript; Y. Y. and R. L. designed the experiments; X. T. and R. L. supervised the whole project. All authors read and approved the manuscript.

## Conflicts of interest

The authors declare no competing financial interest.

## Supplementary Material

NA-004-D2NA00197G-s001
