# Supplementary material for: Rare earth nanoparticles for sprayed and intravenous NIR II imaging and photodynamic therapy of tongue cancer
Source: Nanoscale Adv. 2022 Apr 11;4(9):2224–32. doi: 10.1039/d2na00197g (PMC9418583; doi:10.1039/d2na00197g)
Supplement: NA-004-D2NA00197G-s001 [file NA-004-D2NA00197G-s001.pdf]

Electronic Supplementary Material (ESI) for Nanoscale Advances.
This journal is © The Royal Society of Chemistry 2022

Supplementary Information

Rare earth nanoparticles for spayed and intravenous NIR II imaging with photodynamic therapy for tongue cancer

Lingling Cai[a], Zhan Wang[b], Bi Lin[b], Kaikai Liu[b], Yanxing Wang[b], Ying Yuan[a,*], Xiaofeng Tao[a,*], Ruichan Lv[b,*]

a. Department of Radiology, School of Medicine, Shanghai Ninth People's Hospital, Shanghai Jiao Tong University, Shanghai 200011, China. Email: yuany83@163.com; cjr.taoxiaofeng@vip.163.com.

b. Engineering Research Center of Molecular and Neuro Imaging, Ministry of Education, School of Life Science and Technology, Xidian University, Xi'an, Shanxi 710071, China. Email: rclv@xidian.edu.cn.

1mmol	Gd	Yb	Er	Ce	Eu
1	0.84	0.1	0.01	0.05	0
2	0.86	0.08	0.01	0.05	0
3	0.89	0.05	0.01	0.05	0
4	0.83	0.1	0.02	0.05	0
5	0.85	0.08	0.02	0.05	0
6	0.88	0.05	0.02	0.05	0
7	0.87	0.1	0.02	0	0.01
8	0.89	0.08	0.02	0	0.01
	Gd	Nd	Er	Ce	Eu
9	0.83	0.1	0.02	0.05	0
10	0.73	0.2	0.02	0.05	0

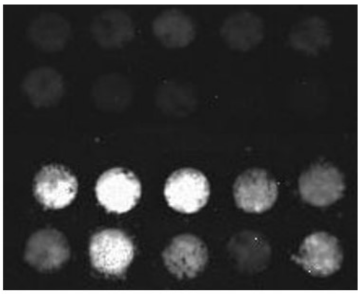

Fig. S1. The components of RENP phosphors in the supplementary first generation and the corresponding NIR II imaging of RENP (808 nm, LP1300).

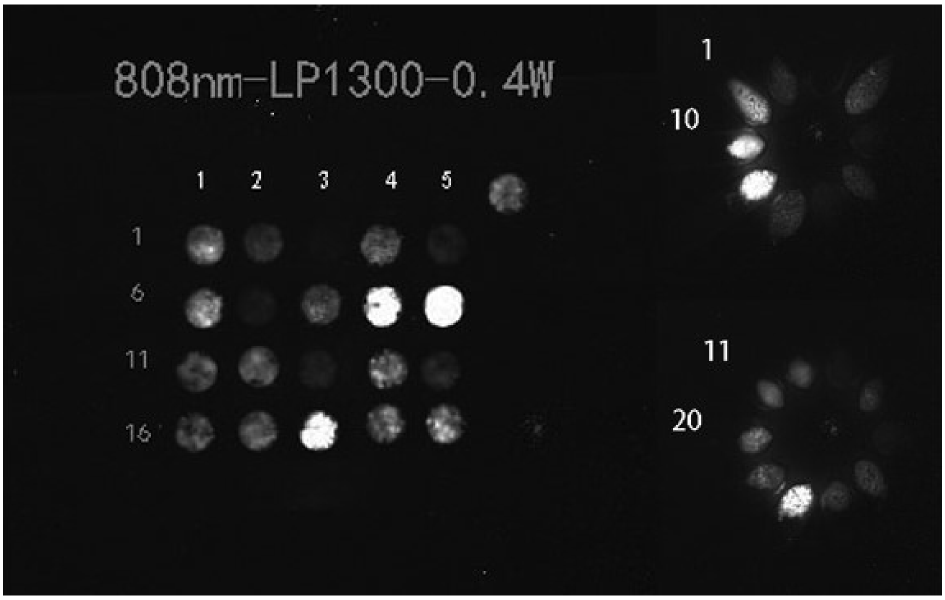

Fig. S2. The NIR II imaging of RENP in the second generation (808 nm, 0.4W, LP1300).

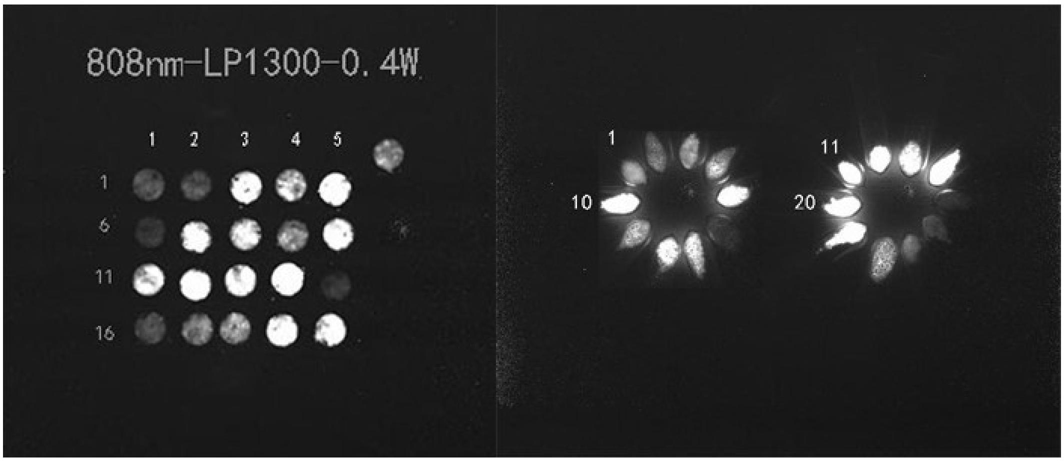

Fig. S3. The NIR II imaging of RENP in the third generation RENP (808 nm, 0.4W, LP1300).

1mmol	Gd	Yb	Er	Ce	Eu
1	0.8	0.123	0.0123	0.0615	0.0032
2	0.67	0.271	0.0271	0.0319	0
3	0.2075	0.6888	0.0454	0.0583	0
4	0.6101	0.339	0.017	0.0339	0
5	0.8648	0.1271	0.0026	0	0.0054
6	0.586	0.3598	0.0237	0.0304	0
7	0.5989	0.294	0.0289	0.0782	0
8	0.723	0.2572	0.0065	0.0133	0
9	0.8695	0.1278	0.0027	0	0
10	0.2872	0.6596	0.0194	0.0338	0
11	0.7534	0.1177	0.0118	0.0589	0.0582
12	0.7009	0.2346	0.0059	0.0586	0
13	0.8624	0.1348	0.0028	0	0
14	0.3558	0.6262	0.0179	0	0
15	0.8645	0.1146	0.0115	0.0095	0
16	0.8465	0.1244	0.0042	0.0249	0
17	0.3599	0.6335	0.0066	0	0
18	0.8439	0.1319	0.0088	0.0154	0
19	0.0996	0.783	0.0391	0.0783	0
20	0.6766	0.2971	0.0088	0.0176	0

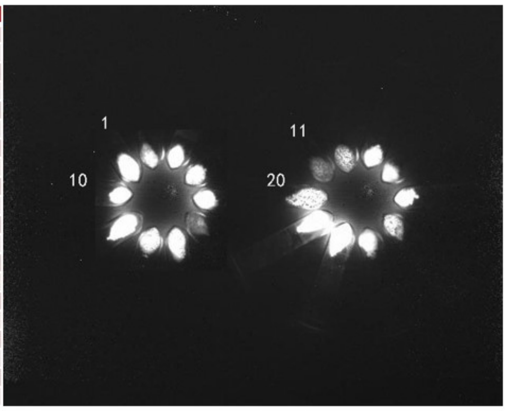

Fig. S4. The NIR II imaging of RENP in the fourth generation RENP (808nm, 0.4W, LP1300).

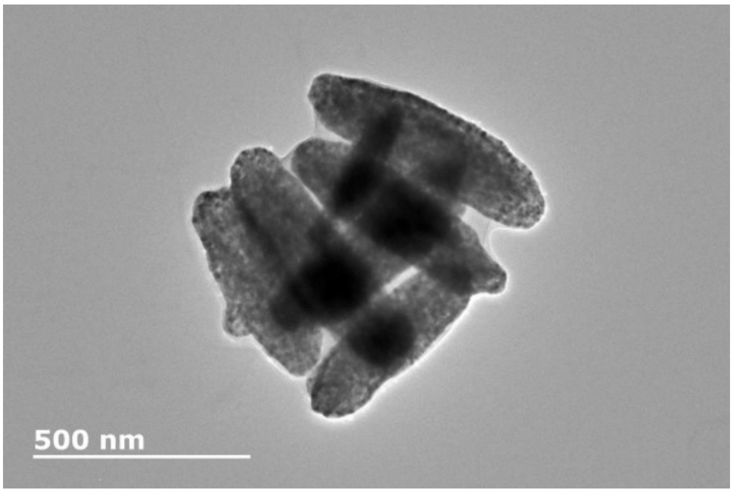

Fig. S5. TEM image of RENP-DHA-Cap.

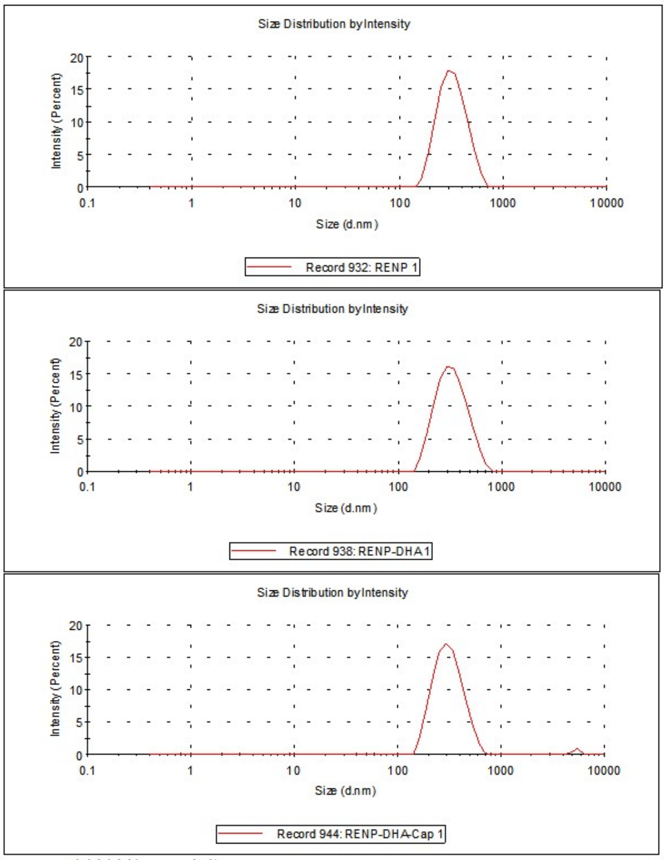

Fig. S6. DLS results of RENP, RENP-DHA, and RENP-DHA-Cap.

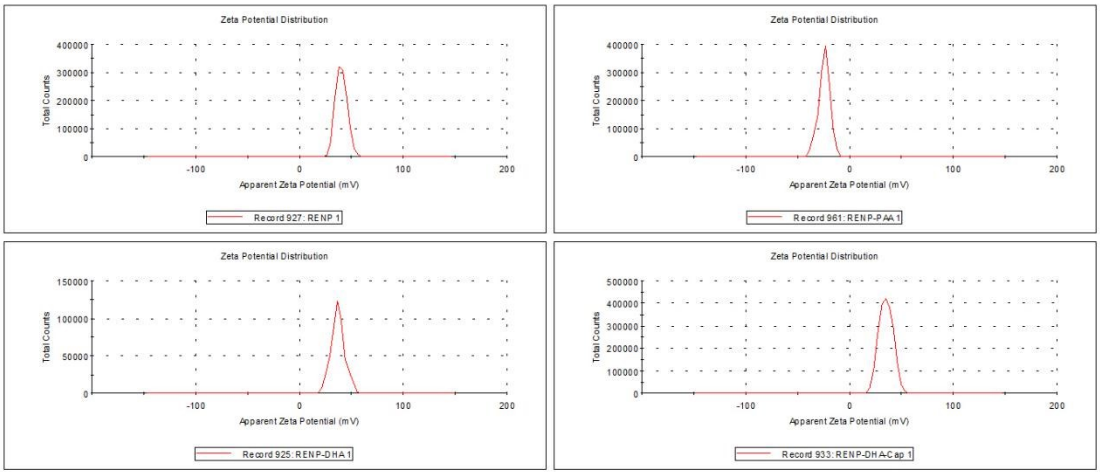

Fig. S7. Zeta potential results of RENP, RENP-DHA, and RENP-DHA-Cap.

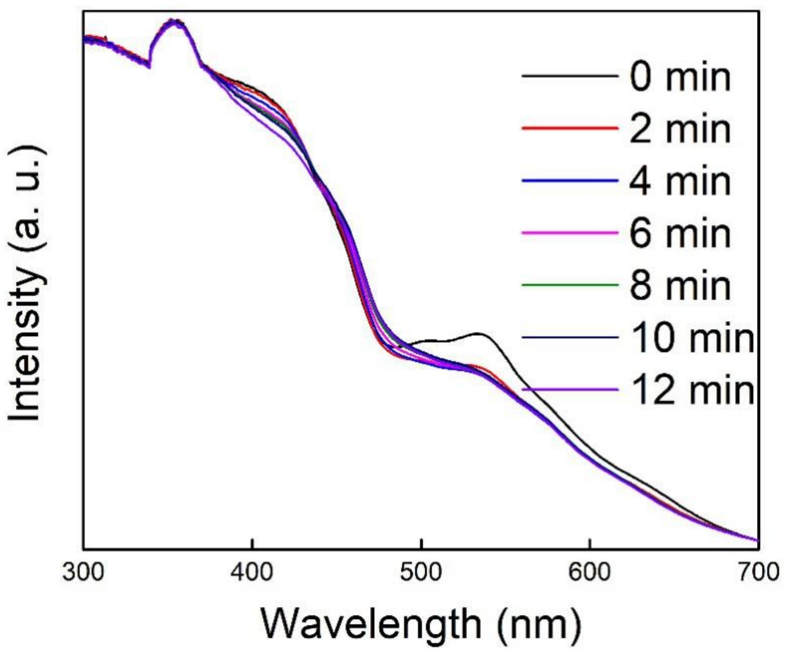

Fig. S8. UV-vis absorbance spectra of DPBF solution together with RENP-Anthocyanin under 980 nm laser with different irradiation time points.

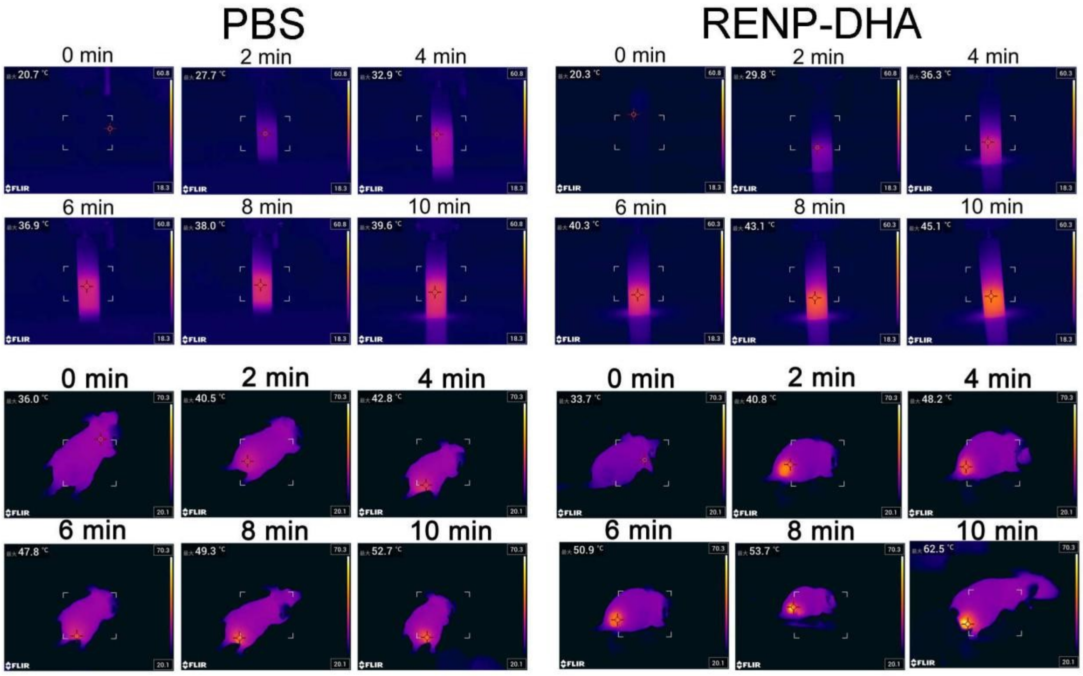

Fig. S9. Infrared thermal photographs of PBS and RENP-DHA at different time points with 980 nm excitation taken by infrared thermal imaging camera.

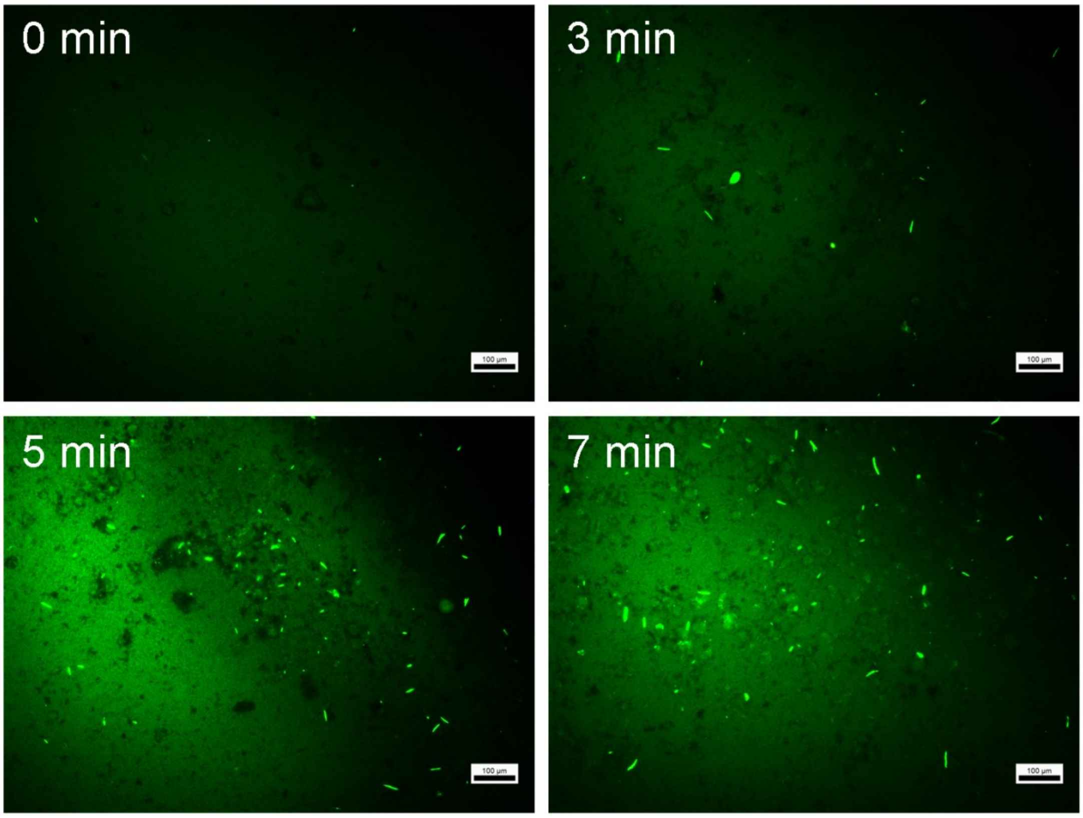

Fig. S10. Microscopy images of Cal 27 cells incubated with RENP-DHA-Cap under 980 nm laser with different irradiation time points. All the cells were finally marked with DCFH-DA.

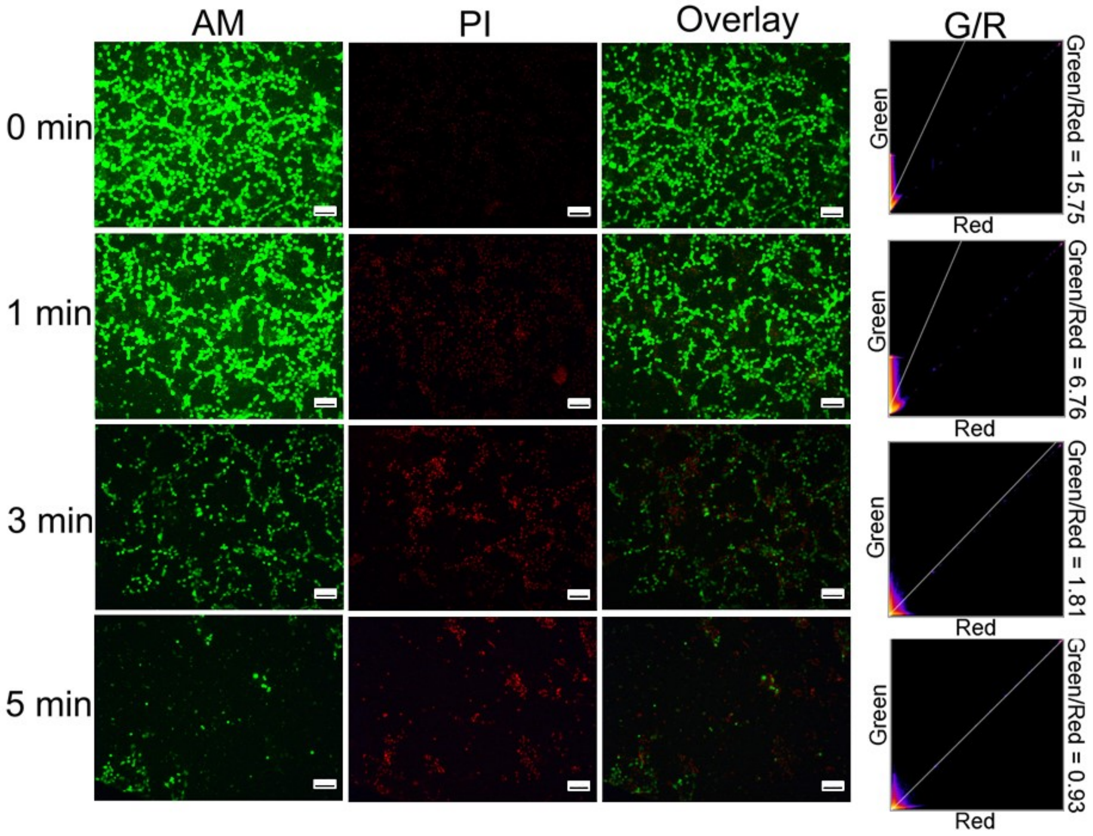

Fig. S11. Microscopy images of Cal27 cells co-incubated with RENP-DHA for 0, 1, 3, 5 min under 980 nm irradiation.

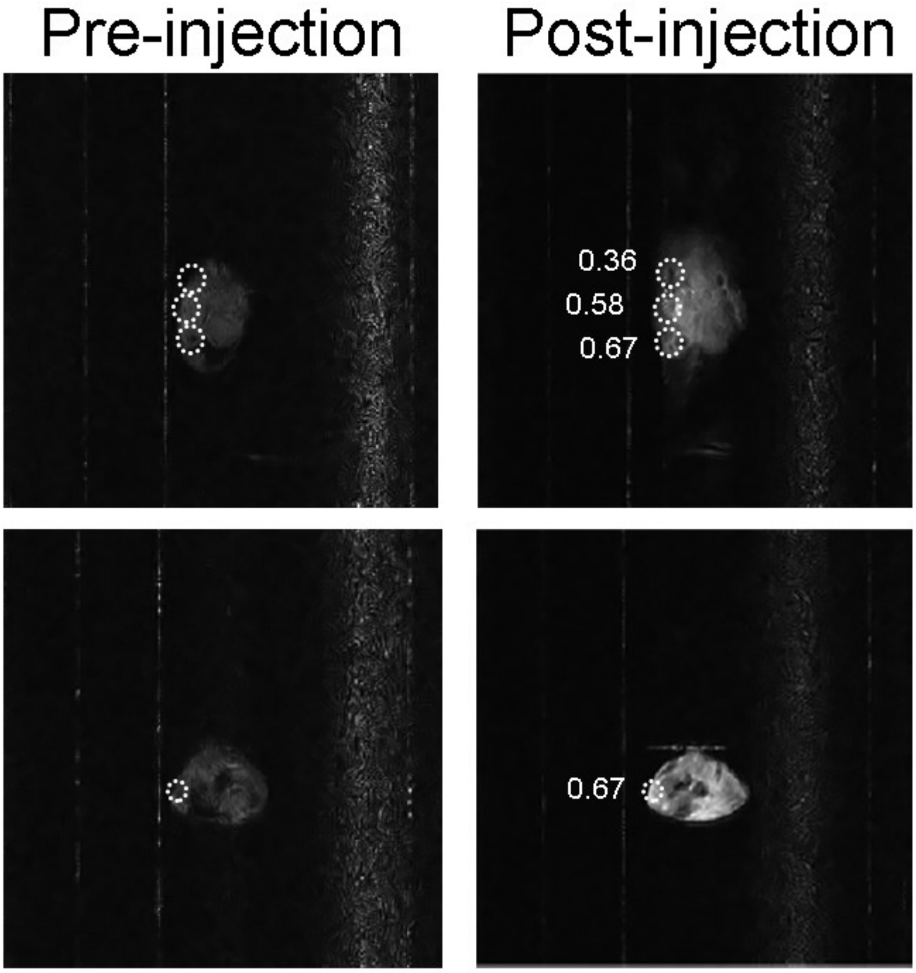

Fig. S12. T_1 signals of MRI in mice using three samples injected subcutaneously (Gd concentrations in RENP: 0.36-0.67; RENP concentration in PBS: 5 mg/mL).

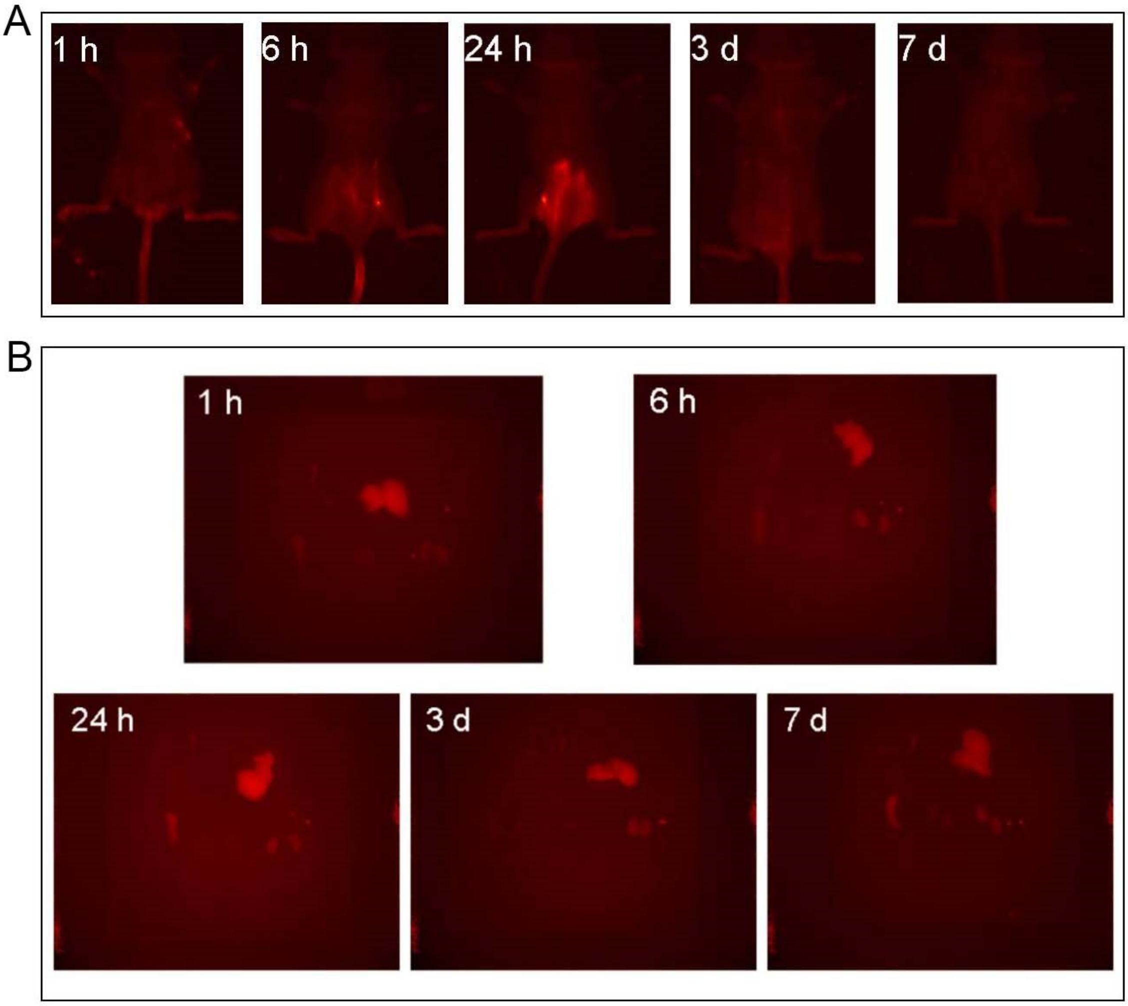

Fig. S13. The *in vivo* NIR II imaging of (A) mice and (B) dissected organs of heart, liver, spleen, lung, and kidney.

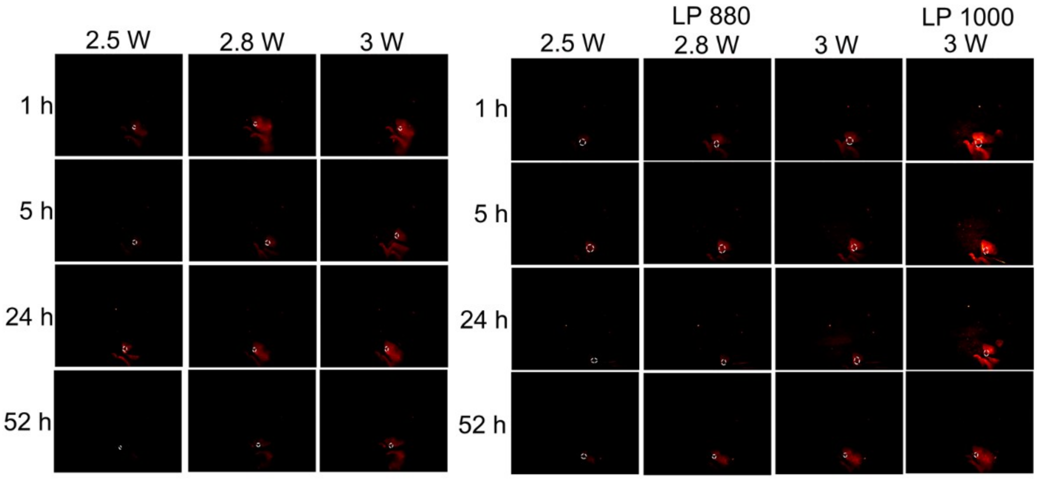

Fig. S14. The NIR II imaging of the mouse with *in situ* tumor after tail vein injection of RENP-DHA-Cap.
